# Differential expression of 12 histone deacetylase (*HDAC*) genes in astrocytomas and normal brain tissue: class II and IV are hypoexpressed in glioblastomas

**DOI:** 10.1186/1471-2407-8-243

**Published:** 2008-08-19

**Authors:** Agda KB Lucio-Eterovic, Maria AA Cortez, Elvis T Valera, Fabio JN Motta, Rosane GP Queiroz, Helio R Machado, Carlos G Carlotti, Luciano Neder, Carlos A Scrideli, Luiz G Tone

**Affiliations:** 1 Department of Pediatrics, Faculty of Medicine of Ribeirao Preto, University of Sao Paulo, Brazil; 2 Department of Genetics, Faculty of Medicine of Ribeirao Preto, University of Sao Paulo, Brazil; 3 Department of Pathology, Faculty of Medicine of Ribeirao Preto, University of Sao Paulo, Brazil; 4 The University of Texas MD Anderson Cancer Center, Department of Neuro-Oncology 6767 Bertner Avenue, BSRB (5th floor), 77030, Houston, Texas, USA

## Abstract

**Background:**

Glioblastoma is the most lethal primary malignant brain tumor. Although considerable progress has been made in the treatment of this aggressive tumor, the clinical outcome for patients remains poor. Histone deacetylases (HDACs) are recognized as promising targets for cancer treatment. In the past several years, HDAC inhibitors (HDACis) have been used as radiosensitizers in glioblastoma treatment. However, no study has demonstrated the status of global *HDAC *expression in gliomas and its possible correlation to the use of HDACis. The purpose of this study was to evaluate and compare mRNA and protein levels of class I, II and IV of HDACs in low grade and high grade astrocytomas and normal brain tissue and to correlate the findings with the malignancy in astrocytomas.

**Methods:**

Forty-three microdissected patient tumor samples were evaluated. The histopathologic diagnoses were 20 low-grade gliomas (13 grade I and 7 grade II) and 23 high-grade gliomas (5 grade III and 18 glioblastomas). Eleven normal cerebral tissue samples were also analyzed (54 total samples analyzed). mRNA expression of class I, II, and IV *HDACs *was studied by quantitative real-time polymerase chain reaction and normalized to the housekeeping gene *β-glucuronidase*. Protein levels were evaluated by western blotting.

**Results:**

We found that mRNA levels of class II and IV *HDACs *were downregulated in glioblastomas compared to low-grade astrocytomas and normal brain tissue (7 in 8 genes, *p *< 0.05). The protein levels of class II HDAC9 were also lower in high-grade astrocytomas than in low-grade astrocytomas and normal brain tissue. Additionally, we found that histone H3 (but not histone H4) was more acetylated in glioblastomas than normal brain tissue.

**Conclusion:**

Our study establishes a negative correlation between *HDAC *gene expression and the glioma grade suggesting that class II and IV *HDACs *might play an important role in glioma malignancy. Evaluation of histone acetylation levels showed that histone H3 is more acetylated in glioblastomas than normal brain tissue confirming the downregulation of *HDAC *mRNA in glioblastomas.

## Background

Gliomas, the most common brain tumor, are currently classified as astrocytic, ependymal, oligodendroglial and choroid plexus tumors. Among astrocytic tumors, glioblastoma (World Health Organization grade IV [1]) is the most lethal primary malignant brain tumor. Although considerable progress has been made in its treatment, the clinical prognosis associated with this tumor remains poor.

**Figure 1:**
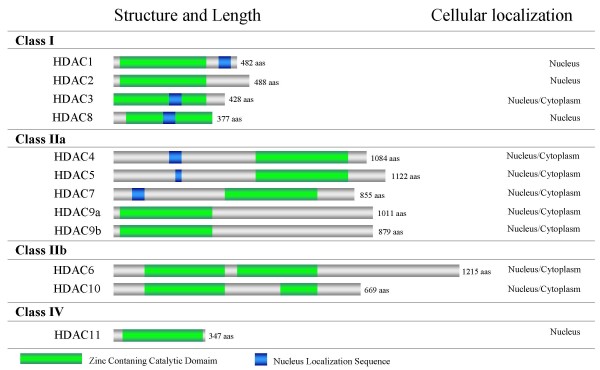
**Classification of classes I, II, and IV HDACs by structure and cellular localization.**[2,6,44,45].

Histone deacetylases (HDACs) have recently become recognized as a promising target for cancer therapy, including for the treatment of glioblastomas [2]. Together with histone acetyltransferases (HATs), HDACs are responsible for chromatin packaging, which influences the transcription process. In general, increased levels of acetylation (high HAT levels) are associated with increased transcriptional activity, whereas decreased acetylation levels (high HDAC levels) are associated with repression of transcription (reviewed in [3]). HDACs are classified into 4 major categories based on their homology to yeast HDACs, including structure and cellular localization (Figure 1). Class I and class II HDAC proteins share a common enzymatic mechanism that is the Zn-catalyzed hydrolysis of the acetyl-lysine amide bond. Human class I HDACs includes HDAC1, -2, -3, and -8, which are enzymes similar to the yeast transcriptional regulator Rpd3, generally localized to the nucleus [4,5]. These enzymes are ubiquitously expressed (with the exception of *HDAC8*, which has higher expression levels in the liver) and seems to function as a complex with other proteins [6]. HDAC1 and -2 only show activity within a protein complex, which consists of proteins necessary for modulating their deacetylase activity and DNA binding, and the recruitment of HDACs to gene promoters [7]. Wilson AJ et al. [8] have suggested that multiple class I HDAC members are also involved in repressing p21 and that the growth inhibitory and apoptotic effects induced by HDAC inhibitors are probably mediated through the inhibition of multiple HDACs.

Class II HDACs includes HDAC4, -5, -6, -7, -9a, -9b, and -10, which are homologous to yeast Hda1. These class II enzymes can be found in the nucleus and cytoplasm, suggesting potential extranuclear functions by regulating the acetylation status of nonhistone substrates [9,10]. HDAC members of class II are abundantly expressed in skeletal muscle, heart, brain, tissues with low levels of mitotic activity [11,12]. Functionally, Class II HDACs is thought to act as transcriptional corepressors by deacetylating nucleosomal histones. These enzymes do not bind directly to DNA; they are thought to be recruited to distinct regions of the genome by sequence specific DNA binding proteins [13-15].

Class III HDACs is composed of the Sirtuins (SIRT) proteins 1–7, which are homologous to the yeast Sir2 protein and require NAD^+ ^for deacetylase activity in contrast to the zinc-catalyzed mechanism used by class I and II HDACs [16-18].

An additional HDAC expressed by higher eukaryotes is a Zn-dependent HDAC (HDAC11 in mammals). This enzyme is phylogenetically different from both class I and class II enzymes and is therefore classified separately as class IV [19] reviewed in [5].

The use of HDAC inhibitors (HDACis) for the treatment of cancer is an area of active investigation. In gliomas, HDACis have been used for the treatment of glioblastoma in combination with radiation therapy and chemotherapy. Some authors have demonstrated that HDACis have a radiosensitizing effect on glioblastoma cells *in vitro *and *in *vivo [20-23] and also seem to be associated with inhibition of glioma cell growth by both cell-cycle arrest and apoptosis [24-26]. Despite the widespread use of HDACis, the mechanistic implications remain to be elucidated.

To this date, there are no studies that demonstrate the status of global HDAC gene expression and protein levels in astrocytomas. The purpose of this study was to evaluate and compare mRNA and protein levels of class I, II and IV of HDACs in low grade and high grade astrocytomas and normal brain tissue and to correlate the findings with the malignancy in astrocytomas.

## Methods

### Patients Samples

For this study, tumor samples of 43 patients (19 men and 24 women) ranging in age from 1.3 to 79 years (mean age 24.6 years, with a median and a standard deviation of 12.8 ± 22.6 years) were evaluated. The histopathologic diagnoses were 20 low-grade gliomas (13 grade I and 7 grade II) and 23 high-grade gliomas (5 grade III and 18 glioblastomas). In addition, 11 samples of normal cerebral tissue were analyzed. Frozen tumor and normal specimens were microdissected. Diagnoses were based on 2007 World Health Organization criteria [1].

For tumor microdissection, tumor samples were placed on a cooled platform and immediately positioned on the cutting base of the cryostat under Tissue Tek (Fisher Scientific, Pittsburg, PA). After rapid freezing in liquid nitrogen, the sample was cut and immediately captured on a coverslip, stained with hematoxylin and eosin, and evaluated by image apposition. The area of interest in the original cryopreserved tumor block was then trimmed, and the microdissected sample was transferred to a previously identified tube, which was immediately placed under dry ice.

Prior to initiation, the research here presented was approved by the Research Ethics Committee of the University Hospital of the Faculty of Medicine of University of Sao Paulo, processes number 9375/2003 and 7645/99. The mentioned Committee is in agreement with the Helsinki Declaration requirements for research carried out on humans. Informed consent was also taken from each patient (or their legal representative) involved in this project, also in accordance to the Helsinki Declaration.

### RNA extraction and cDNA synthesis

Total cellular RNA was extracted using Trizol^® ^Reagent (Invitrogen, Carlsbad, CA, USA) and RNA was reverse transcribed to single-stranded cDNA using a High Capacity Kit (Applied Biossystems, Foster City, CA, USA) according to the manufacturer's protocol.

### Quantitative real-time polymerase chain reaction (qRT-PCR)

Messenger RNA expression level for each *HDAC *was evaluated using an ABI 7500 machine (Applied Biosystems, Foster City, CA, USA). Amplifications were obtained using on demand TaqMan^® ^probes (Applied Biosystems, Foster City, CA, USA) for each *HDAC*. For relative quantification of gene expression, standard curves were constructed for each gene by considering at least 3 points in triplicate of 10-fold serial dilution of cDNA in water, starting from 1:10 of a volume of undiluted cDNA transcribed from 1.0 μg of total RNA. The slopes of standard curves ranged from -3.17 to -3.87. Blank and standard controls (calibrators) were run in parallel to verify amplification efficiency within each experiment. To normalize differences in the amount of total cDNA added to each reaction, *β-glucuronidase *(*GUS β*) gene expression was used as an endogenous control. As a calibrator sample (reference sample for relative quantification), the U343 cell line was used. To obtain the Ct (cycle) values, we established a threshold of 0.1. All reactions were made in duplicate, and all procedures were carried out at 4°C.

### Western Blotting

For protein analysis, 30 μg of each sample was loaded and separated by sodium dodecyl sulphate-polyacrylamide gel electrophoresis [27]. Proteins were transferred to nitrocellulose membranes, and the membranes were then incubated in 1% Tris-buffered saline Tween-20 (TBST) containing 5% (w/v) dried non-fat milk for 1 hour at room temperature. The primary antibodies were diluted at 1:3000 in TBST containing 5% (w/v) milk: HDAC9 (Abcam, Cambridge, MA), Acetyl-Lys H3 (Abcam, Cambridge, MA), and Acetyl-Lys H4 (Upstate Biotechnology Lake Placid, NY), and the membranes were incubated for 1 hour at room temperature, The membranes were then washed 3 times with TBST, incubated with the secondary antibody (1:5000 in TBST) labeled by horseradish peroxidase (Abcam, Cambridge, MA) for 1 hour at room temperature, and washed 3 times with TBST. The secondary antibody was visualized using electron chemiluminescent reagent (Pierce, Rockford, IL). Films were exposed from 10 to 60 seconds and developed.

### Statistical analysis

Comparison of gene expression between groups of tumor was performed by nonparametric testes Mann-Whitney and Kruskall-Wallis. The level of significance was set at *p *< 0.05 in all analyses.

## Results

### Global expression of *HDAC *genes in gliomas and normal brain tissue

**Figure 2:**
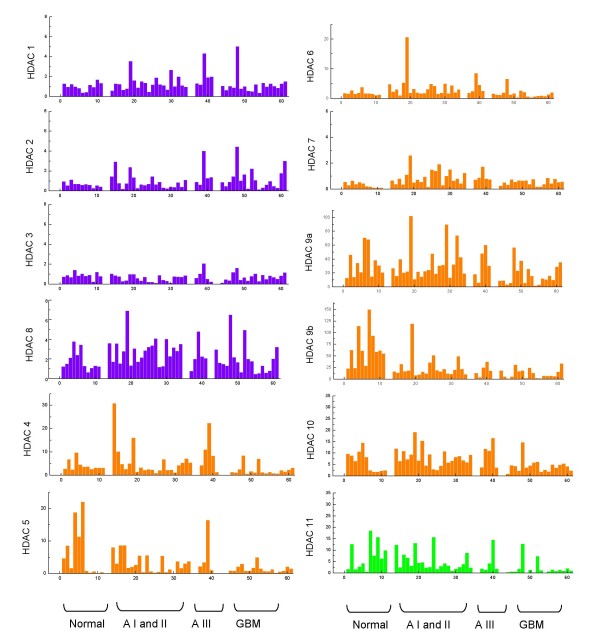
**Relative expression of *HDAC *genes in astrocytomas and normal brain tissue**. Class I in purple, class II in orange, and class IV in green. Data obtained by qRT-PCR analysis using Taqman^® ^probes for each gene using the *GUS β *gene as housekeeping. Standard curves were constructed for each gene by considering at least 3 points in triplicate of 10-fold serial dilution of cDNA in water, starting from 1:10 of a volume of undiluted cDNA transcribed from 1.0 μg of total RNA. The slopes of standard curves ranged from -3.17 to -3.87. Blank and standard controls (calibrators) were run in parallel to verify amplification efficiency within each experiment. To obtain the Ct (cycle) values, we established a threshold of 0.1. *AI, AII and AIII mean astrocytoma grade I, II and III respectively. GBM means glioblastoma.

The expression of 12 *HDAC *genes was analyzed using relative quantification of mRNA levels in normal brain, astrocytomas grades I, II and III, and glioblastomas (Figure 2). Class I *HDAC *genes (*HDAC*1, -2, -3, and -8) showed lower levels of expression (relative expression of 0.5 to 6.0 approximately) compared to the other classes studied. The highest values of expression for class I were seen for *HDAC8 *(2.0 to 6.0). Expression of *HDAC *class II and class IV were higher, with values of approximately 1.0 to 150.0. The highest level of mRNA was observed for *HDAC9a *and *HDAC9b*, with relative expression reaching values above 100 for normal brain tissue and grade I astrocytomas; however, *HDAC6 *and *HDAC7 *showed levels of expression comparable with those of class I (approximately 1.0 and 3.0).

Table 1 shows the median values for each *HDAC *gene in all groups analyzed. For class I, the highest median value observed was 2.87 (*HDAC8 *in low-grade astrocytoma) and the lowest median value was 0.62 (*HDAC3 *in astrocytoma grade III). For class II, the highest median was 61.51 (*HDAC9b *in normal brain) and the lowest was 0.56 (*HDAC7 *in glioblastoma). *HDAC11*, the only member of class IV, had the lowest median value for normal brain tissue (3.67) and the highest for glioblastoma (1.02).

**Table 1:** Relative expression (medians) of *HDAC *genes in different groups of tumor and normal brain tissue.

	**Normal Brain**	**Astrocytoma grades I and II**	**Astrocytoma grade III**	**Glioblastoma**
***Class I***				
*HDAC1*	0.98	1.19	1.93	1.03
*HDAC2*	0.68	0.72	1.30	0.95
*HDAC3*	0.85	0.73	0.62	0.69
*HDAC8*	1.30	2.87	2.08	1.64
				
***Class II***				
*HDAC4*	3.27	3.33	8.43	1.42
*HDAC5*	1.71	2.72	2.27	0.91
*HDAC6*	1.72	3.05	2.57	1.33
*HDAC7*	0.61	0.80	0.81	0.56
*HDAC9a*	30.74	24.46	30.51	11.49
*HDAC9b*	61.51	17.10	18.32	7.43
*HDAC10*	6.54	7.29	11.04	4.26
				
***Class IV***				
*HDAC11*	3.67	3.30	3.05	1.02

### Comparison of the *HDAC *mRNA levels in low- and high-grade gliomas and normal brain tissue

We compared mRNA levels of *HDAC *genes (the medians of relative expression) in low- and high-grade gliomas and also in normal brain. Among class I *HDACs*, significant differences in gene expression between tumor groups were not observed (Table 2).

**Table 2:** Statistical comparison (Kruskall-Wallis analysis) of levels of *HDAC *expression (medians) between low-grade (astrocytomas grade I and II) and high-grade (astrocytomas grade III and glioblastomas) astrocytomas.

	**Low-grade astrocytomas × high-grade astrocytomas ** **(*p *value)**
***Class I***	
*HDAC1*	ns
*HDAC2*	ns
*HDAC3*	ns
*HDAC8*	
	
***Class II***	
*HDAC4*	ns
*HDAC5*	< 0.05
*HDAC6*	< 0.01
*HDAC7*	< 0.05
*HDAC9a*	< 0.01
*HDAC9b*	< 0.0001
*HDAC10*	< 0.001
	
***Class IV***	
*HDAC11*	< 0.001

ns: no significant

Seven of 8 class II *HDAC *genes (exception for *HDAC4*) were expressed at lower levels in high-grade astrocytomas compared to low-grade astrocytomas (*p *< 0.05; Table 2). The same significant difference for low-grade and high-grade gliomas was observed for *HDAC11 *(class IV).

We compared the most malignant form of astrocytoma (glioblastoma) with the other 3 tumors (astrocytomas grades I, II and III) and normal brain in order to establish a correlation between *HDAC *expression and tumor grade (Table 3). As mentioned above, no significant difference in *HDAC *expression was observed for class I; however, for classes II and IV, there was a decrease in expression of these genes in glioblastoma compared to that in other groups. Moreover, this downregulation appeared to follow a pattern in which lower-grade tumors had a larger number of *HDAC *genes at lower expression levels. Comparison between glioblastoma and grade III astrocytoma showed that 4 of 8 genes were expressed at lower levels in glioblastoma samples (*HDAC4*, *-6*, *-7*, *and -11*). Comparison with low-grade astrocytoma (grades I and II) showed that the expression of 6 of 8 genes was lower in glioblastomas (except for *HDAC5 *and -*7*). Finally, when we compared glioblastoma with normal brain, 7 of 8 genes studied, with the exception of *HDAC7*, were expressed at lower levels in glioblastoma.

**Table 3:** Statistical comparison (Mann-Whitney analysis) of levels of *HDAC *expression (medians) between tumor groups.

	*p *value			
	
	*HDAC1*	*HDAC2*	*HDAC3*	*HDAC8*
Glioblastoma x				
Normal brain	ns	ns	ns	ns
AI and II	ns	ns	ns	ns
AIII	ns	ns	ns	ns
	
	*HDAC4*	*HDAC5*	*HDAC6*	*HDAC7*

Glioblastoma x				
Normal brain	<0.0001	0.018	0.011	ns
AI and II	0.012	ns	0.001	ns
AIII	0.010	ns	0.010	0.008
	
	*HDAC9a*	*HDAC9b*	*HDAC10*	*HDAC11*

Glioblastoma x				
Normal brain	0.0096	<0.0001	0.015	0.0001
AI and II	0.01	0.013	0.001	0.0003
AIII	ns	ns	ns	0.023

AI, AII, and AIII mean astrocytoma grades I, II and III, respectively.

ns: no significant

### Protein analysis: Acetyl H3 but not Acetyl H4 correlates with mRNA levels

**Figure 3:**
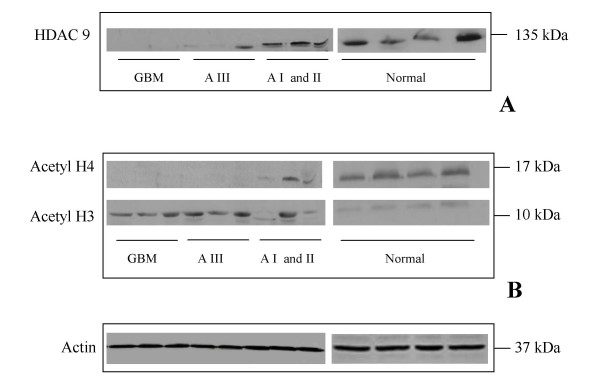
**Protein analysis**. (A) HDAC9 and (B) acetyl H3 and acetyl H4 in glioblastoma (GBM), astrocytoma grade III (AIII), astrocytoma grades I and II (AI and II), and normal brain. Actin was used as endogenous control.

In order to validate the data obtained from qRT-PCR, western blot analysis was performed for HDAC9b protein. This protein was chosen because we found the highest levels of mRNA expression for HDAC9b. The results obtained for HDAC9b western blot analysis confirmed the data obtained in quantitative mRNA analysis. The protein levels of HDAC9a were higher in normal brain tissue and low-grade astrocytoma than in the grade III astrocytoma and glioblastoma (Figure 3A).

Anti-acetyl histone H3 and anti-acetyl histone H4 antibodies were also used to verify the level of acetylated histones H3 and H4 and to correlate the findings with histone deacetylase activity in the groups studied (Figure 3b).

Considering the large number of *HDAC *genes with low levels of expression in glioblastomas, we expected that the levels of acetylated histones were higher in those tumors. Interestingly, when we analyzed the acetylation levels of the H3 and H4 histones, H3 histone acetylation, but not H4 histone acetylation, correlated with the data obtained by qRT-PCR (Figure 3B). Glioblastoma samples showed higher levels of acetylated histone H3 than normal brain and low-grade gliomas.

## Discussion

In this study we evaluated and compared mRNA levels of 12 *HDAC *genes in astrocytomas and normal brain tissue. As mentioned before, the acetylation levels of histones is a process regulated by two groups of important enzymes: HATs and HDACs, and the balance of the activities of these two enzymes is tightly related to the gene expression status in the cell. The regulation of HDACs has been studied, but is not yet well established. The reason for that is maybe because HDACs seem to be regulated at multiple levels including cellular compartmentalization, association with other factors, abundance, activity states and genome-wide distribution.

Considering that altered gene expression is frequently observed in cancer, a relationship between HDACs and cancer progression has been postulated. Wade P. [3] suggested that loss of targeting of class I HDACs through disruption of a transcriptional corepressor and the inappropriate redistribution of class II resulted in the misregulated gene expression in cancer.

Although a crucial role for HDACs in gene transcription and their possible involvement in cancer has been proposed, no studies have demonstrated the expression profile of 3 classes of HDACs simultaneously in brain tumor.

Our study did not find differential gene expression of class I *HDACs *in high-grade and low-grade gliomas which may indicate that class of deacetylases seems to not be directly involved with malignancy of gliomas. Only a few studies have evaluated the level of class I *HDAC *expression in cancer: Huang BH et al. [28] demonstrated that *HDAC1 *and *HDAC2 *seem to be upregulated in colon cancer; Choi JH et al. [29] demonstrated an overexpression of *HDAC1 *mRNA 68% of gastric cancer tissues studied by them (17 of 25) and elevated expression of HDAC1 protein was also detected in 61% of the gastric cancer samples (11 of 18). Expression of class I *HDAC3 *was also shown elevated in astrocytic glial tumors compared to nonmalignant gliosis [30]. On the other hand, Ozdag H. et al. [31] showed that *HDAC1 *is significantly lower in colorectal cancer samples in comparison to normal colorectal tissues.

For class II *HDACs*, downregulation of its expression in glioblastoma compared to low-grade gliomas and normal brain tissue was demonstrated and statistically confirmed in our study, indicating a negative correlation between *HDAC *expression and malignancy in gliomas. In agreement with our study, some authors have demonstrated downregulation of these deacetylases and their relationship with prognosis in cancer as well. Ozdag H. et al. [31] showed that *HDAC5 *and *HDAC7 *are significantly lower in colorectal cancer samples in comparison to normal colorectal tissues. These authors also showed downregulation of *HDAC5 *in renal tumors compared to normal renal tissue. Downregulation of *HDAC *gene expression has also been observed in lung cancer: reduced expression of class II *HDAC *genes (mostly *HDAC10*) seems to be significantly associated with poor prognosis, suggesting that class II *HDACs *may repress critical genes that may have important roles in lung cancer progression [32]. Like in our study, these authors demonstrated that class I *HDACs *seem to have no correlation with malignancy of those tumors.

The downregulation of *HDAC *genes in cancer may be difficult to explain, since multiple factors are involved in the regulation of these enzymes. Unlike class I HDACs, which are predominantly localized in the nucleus, class II HDACs actively shuttle between the cytoplasm and nucleus being under control of classic cellular signaling pathways, and cellular localizations represents a fundamental mechanism for them [12,14]. Additionally, class II *HDACs *seem to have additional levels of regulation, which makes elucidation of the mechanism of gene transcription regulation more complicated.

Our study seems to reveal the involvement of class II HDAC*s *in glioma malignancy. If we consider that, in general, increased levels of acetylation (downregulation of HDACs) is related to higher transcriptional activity, we could predict that gene transcription in malignant gliomas may be upregulated. In that case we could infer that some proto-oncogenes might be overexpressed and somehow leading to the malignancy. The overexpression of proto-oncogenes in gliomas has been well documented. Besides *EGFR*, which is found overexpressed in 40% of gliomas, the genes *N-MYC*, *C-MYC*, *PDGFR-α, MYB*, *K-RAS*, *CDK*-4 and *MDM*2 are the most commonly amplified oncogenes in gliomas [33-37]. However, due to the fact that these deacetylases are regulated at several levels, additional information about the functionality of HDACs in gliomas is required.

In order to correlate mRNA and protein levels of HDACs, we analyzed HDAC9 protein levels in all groups of tumors studied. Western blot analysis showed that HDAC9 protein is expressed at a higher concentration in normal brain and low-grade gliomas than in high-grade gliomas, validating the data obtained by real-time PCR.

We also evaluated HDAC activity by analyzing histone acetylation levels in tumor samples and normal brain. When we analyzed the levels of H3 and H4 acetylated histones, we observed an increased level in acetylation of H3 but not of H4 histone in glioblastomas compared to low-grade astrocytomas and normal brain tissue. Considering the low levels of *HDAC *expression in glioblastomas, it was expected that the levels of acetylated histones should be more elevated in those tumors, as demonstrated here for histone H3. On the other hand, the lack of correlation between low *HDAC *expression and high histone H4 deacetylation levels in glioblastomas could be explained by the existence of cofactors or unidentified regulators. Some few authors have already demonstrated that the specificity of HDACs depends on cofactors which makes HDAC specificity a complicated process [38-41]. It is tempting to speculate that class II of HDACs could be responsible for deacetylation of histone H3 more than histone H4. However, the data here presented are not enough to infer about the specificity of HDACs in astrocytomas. Moreover, it has been observed that differences between HDAC subtype specificity do not coincide with the division into class I and class II enzymes. HDAC1, HDAC3 (class I), and HDAC6 (class II) seem to be very similar in substrate specificity and mainly differ in the degree of specificity [39]. Until now, a lot of data has been generated about HDAC research, but the natural substrates of different HDACs and their substrate specificities is still not well understood.

The class IV HDAC11 enzyme seems to be an unusual member of the HDAC family. Its sequence is not homologous to any other HDAC, and it may have distinct physiological roles. This enzyme, like members of the class II HDACs is expressed more in brain, heart, skeletal muscle, and kidney [19]. In our study, we also found high levels of *HDAC11 *in normal brain tissue and significant differential expression was also observed for this enzyme in low-grade and high-grade gliomas. Little is known about this unique HDAC, and its relation to malignancy of gliomas is yet to be elucidated.

### HDACis

Even considering that to evaluate the effect of HDAC inhibitors in gliomas was not the aim of the present study, the finding that most of the *HDAC *genes are downregulated in glioblastomas could lead us to consider that HDACis may not be effective in the treatment of high-grade gliomas. Some studies have demonstrated radiosensitization of gliomas after HDACi treatment [21,22]. Therefore, despite the low levels of *HDAC *gene expression in glioblastomas, HDACis seem to be potential therapeutical targets for glioma treatment. The explanation to this may point to the existence of nonhistone substrates for HDACs. Although histones are the most thoroughly studied as HDAC substrates, several reports have shown that HDACs are also responsible for the deacetylation of diverse types of nonhistone proteins, including transcriptional factors, signal transduction mediators, and molecular chaperones (a summarized table of nonhistone substrates for HDACs can be found in [2]). Additionally, recent evidences suggest that modulation of gene expression through histone remodeling might not be the only process responsible for the antiproliferative action of HDACis [42,43]. Although the present study has demonstrated that most of *HDAC*s genes are downregulated in glioblastomas, no experiment was performed in order to analyze the effect of HDAC inhibitors on glioma treatment, therefore the effect of these inhibitors on glioma treatment should be addressed in a separate manuscript.

## Conclusion

Our study has established a negative correlation between *HDAC *gene expression and glioma grade, suggesting that class II and class IV *HDACs *might play an important role in glioma malignancy. This differential *HDAC *expression may provide insight into development of novel treatment approaches for this devastating disease. However, a more complete understanding of the biological function and specificity of the diverse HDAC isoforms and their involvement in the cancer process is necessary.

## Competing interests

The authors declare that they have no competing interest.

## Authors' contributions

Experiments and collection of data were performed by AKBL-E, MAAC, ETV, FJNM and RGPQ. Collection of tumor samples was performed by HRM and CGCJr. Microdissection of the tumor samples was performed by LN. AKBL-E was responsible for data analysis and interpretation and also wrote the manuscript. Manuscript reviewing was made by AKBL-E, MAAC, CAS and LGT.

## Pre-publication history

The pre-publication history for this paper can be accessed here:


